# Comparative milk proteome analysis of Kashmiri and Jersey cattle identifies differential expression of key proteins involved in immune system regulation and milk quality

**DOI:** 10.1186/s12864-020-6574-4

**Published:** 2020-02-14

**Authors:** Shakil A. Bhat, Syed M. Ahmad, Eveline M. Ibeagha-Awemu, Mohammad Mobashir, Mashooq A. Dar, Peerzada T. Mumtaz, Riaz A. Shah, Tanveer A. Dar, Nadeem Shabir, Hina F. Bhat, Nazir A. Ganai

**Affiliations:** 1 Division of Animal Biotechnology, Faculty of Veterinary Sciences and Animal Husbandry, SKUAST-Kashmir, Srinagar, India; 2 Agriculture and Agri-Food Canada, Sherbrooke Research and Development Centre, Sherbrooke, Quebec Canada; 3 Department of Microbiology, Tumor and Cell Biology (MTC), Karolinska Institute, Novels väg 16, 17165 Solna, Stockholm, Sweden; 4 Department of Clinical Biochemistry, University of Kashmir, Srinagar, J & K India; 5 Division of Animal Genetics and Breeding, Faculty of Veterinary Sciences and Animal Husbandry, SKUAST-Kashmir, Srinagar, India

**Keywords:** Jersey, Kashmiri, Milk proteome, FMO3 enzyme

## Abstract

**Background:**

Exploration of the bioactive components of bovine milk has gained global interest due to their potential applications in human nutrition and health promotion. Despite advances in proteomics profiling, limited studies have been carried out to fully characterize the bovine milk proteome. This study explored the milk proteome of Jersey and Kashmiri cattle at day 90 of lactation using high-resolution mass spectrometry based quantitative proteomics nano-scale LC-MS/Q-TOF technique. Data are available via ProteomeXchange with identifier PXD017412.

**Results:**

Proteins from whey were fractionated by precipitation into high and low abundant proteins. A total of 81 high-abundant and 99 low-abundant proteins were significantly differentially expressed between Kashmiri and Jersey cattle, clearly differentiating the two breeds at the proteome level. Among the top differentiating proteins, the Kashmiri cattle milk proteome was characterised by increased concentrations of immune-related proteins (apelin, acid glycoprotein, CD14 antigen), neonatal developmental protein (probetacellulin), xenobiotic metabolising enzyme (flavin monooxygenase 3 (FMO3), GLYCAM1 and HSP90AA1 (chaperone) while the Jersey milk proteome presented higher concentrations of enzyme modulators (SERPINA1, RAC1, serine peptidase inhibitor) and hydrolases (LTF, LPL, CYM, PNLIPRP2). Pathway analysis in Kashmiri cattle revealed enrichment of key pathways involved in the regulation of mammary gland development like Wnt signalling pathway, EGF receptor signalling pathway and FGF signalling pathway while a pathway (T-cell activation pathway) associated with immune system regulation was significantly enriched in Jersey cattle. Most importantly, the high-abundant FMO3 enzyme with an observed 17-fold higher expression in Kashmiri cattle milk seems to be a characteristic feature of the breed. The presence of this (FMO3) bioactive peptide/enzyme in Kashmiri cattle could be economically advantageous for milk products from Kashmiri cattle.

**Conclusion:**

In conclusion, this is the first study to provide insights not only into the milk proteome differences between Kashmiri and Jersey cattle but also provides potential directions for application of specific milk proteins from Kashmiri cattle in special milk preparations like infant formula.

## Background

Bovine milk is a valued natural product which delivers a matrix of essential nutrients including growth and immune factors to offspring and a key raw material for human food preparations [1, 2]. Some studies have characterized the bovine milk proteome, its bioactive profile, and the extent of cross reactivity of bovine bioactive milk peptides on various biological functions [3–7]. Milk proteins are generally categorized into three major groups: caseins, whey proteins and milk fat globule membrane proteins [4, 8]. Most of the polypeptides in milk are an essential source of amino acids to neonates [9] and many resist proteolysis [10, 11]. Milk peptides also facilitate absorption of other nutrients in the gastro-intestinal tract, provide humoral immune responses and support intestinal development [12]. Besides, digestion or fermentation of milk proteins also produces a number of bioactive peptides, which contribute as well to the various functional properties of milk [13, 14]. The major proteins in milk are far outnumbered by numerous other minor proteins which play important roles in a wide range of physiological activities including antioxidant activity, post-natal development of new-borns, maturation of the immune system, establishment of symbiotic microflora, and protection against various pathogens [15, 16].

Several studies have characterised the milk proteome in different species and breeds using different quantitative proteomic techniques [7, 16–20]. The differences in the milk proteome profile have been attributed to genetic, management and disease factors [7, 21]). Although the diverse composition and biological functions of bovine milk has been reported extensively [22–24], the comparative abundance of milk proteins in Indian cattle breeds have not been investigated till date. Kashmiri and Jersey cattle are two important milk animals which contribute significantly to the total milk production in the Indian northern state of Kashmir. The Kashmiri cattle is an indigenous breed kept mainly for milk production in the hilly regions of Kashmir. Kashmiri cattle are small, hardy and adapted to the hilly regions of Kashmir. Whereas, Jersey is a well-established dairy breed imported to augment the milk production ability of Kashmiri cattle through cross breeding. We hypothesize that the proteome profile of Kashmiri cattle milk may have special properties or differ from that of the well-established Jersey dairy breed due to its different genetic background and milk producing ability. Therefore, the aim of this study was to study the protein profiles of Kashmiri and Jersey cattle milk which could reveal important protein factors underlying the physiological differences and differences in milk traits between the two breeds.

## Results

### Proteome profile of bovine milk

**Fig. 1:**
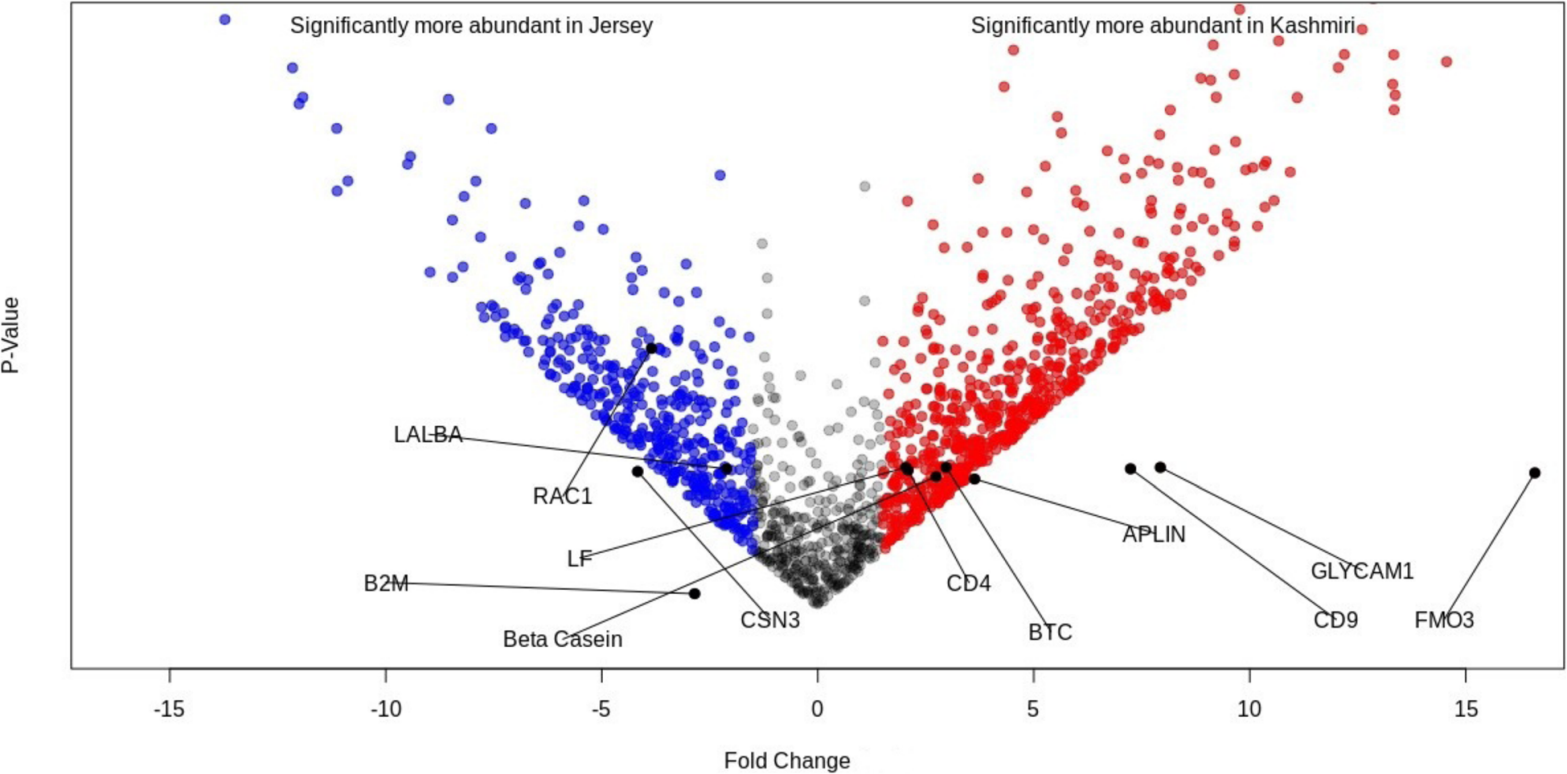
Volcano plot of differentially expressed proteins between Kashmiri and Jersey cattle. Red points indicate more abundant proteins in Kashmiri cattle; blue points indicate more abundant proteins in Jersey cattle

Proteins from whey were fractionated by precipitation into high and low abundant proteins. A total of 180 proteins were differentially expressed (DE) (FDR < 0.1) between Kashmiri and Jersey cattle. Specifically, 91 and 89 proteins were significantly upregulated (FDR < 0.1) in Kashmiri and Jersey cattle, respectively (Additional file 2: Table S2a and S2b, Additional file 3). The most upregulated high abundant proteins (fold change (FC) > 2) were CSN2, CD4 and LF, and low abundant proteins were FMO3, GLYCAM1, APLN and BTC in Kashmiri cattle (Table 1, Fig. 1). Whereas, LALBA, ZNF496, CSN3 and LGB were the most upregulated high abundant proteins and RAC1, B2M and SAR1B were the most upregulated minor milk proteins in Jersey cattle (Table 1).

**Table 1:** Significantly upregulated high abundant and low abundant milk proteins in Kashmiri and Jersey cattle

	Accession No.	Protein	Gene ID	FC	*P*-value	FDR
Kashmiri Cattle	Significantly upregulated abundant milk proteins					
	J9UHS4	Beta-casein	CSN2	2.74	0.044	0.055
	Q8HY42	CD4 antigen	CD4	2.09	0.039	0.043
	E6YCQ7	Lactoferrin	LF	2.04	0.037	0.047
	Significantly upregulated less abundant milk proteins					
	Q8HYK4	Flavin-containing monooxygenase 3	FMO3	16.6	0.041	0.050
	P80195	Glycosylation-dependent cell adhesion molecule 1	GLYCAM1	7.93	0.037	0.047
	P30932	CD9 antigen	CD9	7.24	0.038	0.048
	Q9TUI9	Apelin	APLN	3.63	0.046	0.050
	Q9TTC5	Probetacellulin	BTC	2.97	0.037	0.042
	Q9TRC0	Enterotoxin-binding glycoprotein PP16K	N/A	2.91	0.038	0.048
	Q3SZR3	Alpha-1-acid glycoprotein	ORM1	2.66	0.046	0.050
	C4PU73	Serin peptidase inhibitor, clade A	LOC286871	2.53	0.039	0.046
	Q9TS52	Adipocyte differentiation-related protein	N/A	2.53	0.042	0.055
	P46201	Uterine milk protein	N/A	2.41	0.043	0.049
	Q5GN72	Alpha-1-acid glycoprotein	AGP	2.07	0.037	0.040
Jersey cattle	Significantly upregulated abundant milk proteins					
	P02754	Beta-lactoglobulin	LGB	7.24	0.037	0.051
	A0A140T8A9	Kappa-casein	CSN3	4.17	0.04	0.046
	F6I8C5	Zinc finger protein 496	ZNF496	2.33	0.037	0.061
	G9G9X6	Alpha-lactalbumin	LALBA	2.11	0.038	0.041
	Significantly upregulated less abundant milk proteins					
	G8FZ88	ATP synthase subunit A	N/A	4.09	0.037	0.047
	P62998	Ras-related C3 botulinum toxin substrate	RAC1	3.85	0.044	0.067
	P01888	Beta-2-microglobulin	B2M	2.85	0.039	0.041
	Q3T0T7	GTP-binding protein SAR1b	SAR1B	2.2	0.037	0.046
	Q9XSC9	Transcobalamin-2	TCN2	2.18	0.044	0.051
	Q95114	Lactadherin	MFGE8	2.11	0.039	0.049

### Enriched gene ontology terms of significantly upregulated proteins in Kashmiri and Jersey cattle

Gene ontology (GO) enrichment of significantly upregulated proteins in Kashmiri and Jersey cattle found a total of 4 enriched GO terms in Kashmiri and 4 in Jersey cattle (Table 2). Only extracellular region (GO:0005576) reached significance after FDR correction in both breeds (Table 2).

**Table 2:** Gene ontology terms enriched for significantly upregulated proteins in Kashmiri and Jersey cattle

Functions	Description	GO term	No. of proteins	Protein IDs	Gene IDs	P-value	FDR
Kashmiri Cattle							
Molecular	Catalytic activity	GO:0003824	18	P19120, P12763, P30122, P11017, Q8MK44, P80209, P62871, Q8HXQ5, Q4GZT4, Q0IIG8, A5PK46, P00794, P80929, Q0VCZ8, Q2UVX4, P80025	HSPA8, AHSG, CEL, GNB2, DGAT1, CTSD, GNB1, ABCC1, ABCG2, RAB18, PNLIPRP2, CYM, ANG2, ACSL1, C3, LPO	0.0002	0.44
	Antioxidant activity	GO:0016209	1	P80025	LPO	0.0843	0.79
Cellular	Membrane	GO:0016020	4	P19120, P30122, Q8MK44, P80209, Q8HXQ5, Q4GZT4, Q0IIG8, P00794, P02702, P30932, P18892	HSPA8, CEL, DGAT1, CTSD, ABCC1, ABCG2, RAB18,CYM,FOLR1,CD9,BTN1A1	0.0198	0.181
	Extracellular region	GO:0005576	10	P46201, P02666, P30122, C4PU73, Q9TTE1, P21214, P80025	Uterine milk protein, CSN2, CEL, Serin peptidase inhibitor,SERPINA3–1,TGFB2,LPO	0.00111	0.0354
Jersey Cattle							
Molecular	Reproduction	GO:0000003	2	A0A140T8A9, P11151, P02668, A5PK46	CSN3, LPL, CSN3,	0.005	0.422
	Catalytic activity	GO:0003824	22	Q8HYJ9, Q5E9R3, P11151, Q5E9B1, Q8MK44, P62998, Q2TBH2, Q8HXQ5, Q148J4, F1MN60, P101, Q4GZT4, A5PK46, P00794, P80457, P02754, P80025	FMO3, EHD1, LPL, LDHB, DGAT1, RAC1, RRAS, ABCC1, RAB2A, ATP2B2, ANG1, ABCG2, PNLIPRP2, CYM, XDH, LGB, LPO	0.066	0.83
	Antioxidant activity	GO:0016209	1	P80025	LPO	0.087	0.52
Cellular	Extracellular region	GO:0005576	11	P17697, A0A140T8A9, P34955, P46201, P02663, Q3ZCH5, P41361, P28800, C4PU73, P02662,P02668,P21214,P80025	CLU, CSN3, SERPINA1, Uterine milk protein, CSN1S2, AZGP1, SERPINC1, SERPINF2, Serin peptidase inhibitor, CSN1S1, CSN3, TGFB2, LPO	0	0

### Protein categories identified through GO annotation

**Fig. 2:**
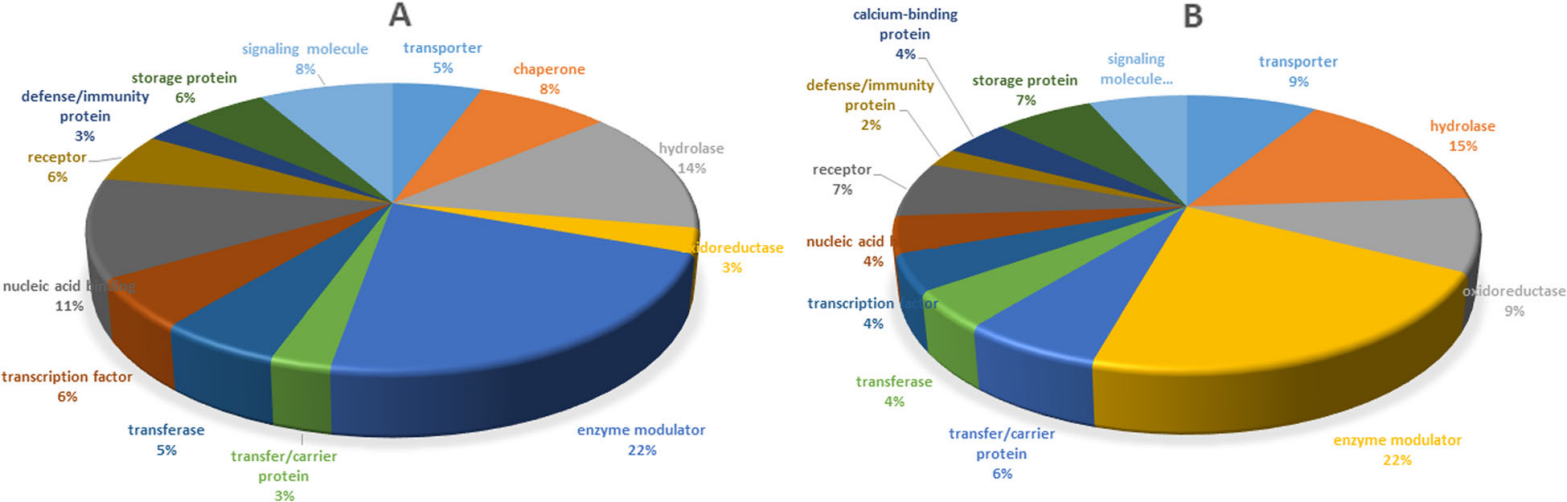
Classification of differentially expressed proteins in Kashmiri and Jersey cattle by gene ontology annotation (**a**) Protein classes (upregulated proteins only) in Kashmiri cattle and (**b**) Jersey cattle

The identified differentially upregulated proteins in Kashmiri and Jersey cattle were categorized according to their GO annotation (Additional file 2: Table S103). Most of the significantly upregulated proteins in both cattle breeds were enzyme modulators (SERPINA3, BTN1A1, SERPINC1, SERPINF2, Serin peptidase inhibitor, RAC1, RRAS, BTN1A1 and uterine milk protein) and hydrolases (GNB2, CTSD, GNB1, PNLIPRP2, CYM) (Fig. 1 a and b). However, proteins belonging to the chaperone classes (HSP90AA1, YWHAB, YWHAZ) were significantly upregulated in Kashmiri cattle only (Fig. 2a and b).

### Enriched pathways by significantly upregulated proteins in Kashmiri and Jersey cattle

Significantly upregulated proteins in Kashmiri and Jersey cattle were enriched to 12 and 4 pathways at uncorrected *P* < 0.05, respectively (Table 3). When FDR correction was applied, 10 and one proteins remained significant (FDR < 0.1) in Kashmiri and Jersey cattle, respectively (Table 3). Of all the pathways, only EGF receptor signalling pathway was enriched at uncorrected P < 0.05 by significantly upregulated proteins in both breeds.

**Table 3:** Enriched pathways by upregulated proteins in Kashmiri and Jersey cattle

Pathway	Proteins	p-value	FDR	Proteins	Genes
Kashmiri cattle					
Beta3 adrenergic receptor signaling pathway (P04379)	2	0.07265	0.1719	P11017, P62871	GNB2, GNB1
Beta2 adrenergic receptor signaling pathway (P04378)	2	0.09576	0.8552	P11017, P62871	GNB2, GNB1
Metabotropic glutamate receptor group III pathway (P00039)	2	**0.0124**	**0.0723**	P11017, P62871	GNB2, GNB1
Beta1 adrenergic receptor signaling pathway (P04377)	2	**0.00599**	**0.0543**	P11017, P62871	GNB2, GNB1
5HT4 type receptor mediated signaling pathway (P04376)	2	0.07387	0.1987	P11017, P62871	GNB2, GNB1
5HT2 type receptor mediated signaling pathway (P04374)	2	**0.0141**	**0.0792**	P11017, P62871	GNB2, GNB1
5HT1 type receptor mediated signaling pathway (P04373)	2	0.09671	0.1897 **0.049**	P11017, P62871	GNB2, GNB1
Integrin signalling pathway (P00034)	2	0.0823	0.353	P63258, E1BBG2	ACTG1, MICALL1
Heterotrimeric G-protein signaling pathway-Gi alpha and Gs alpha mediated pathway (P00026)	2	0.0658	0.298	P11017, P62871	GNB2, GNB1
Wnt signaling pathway (P00057)	3	**0.0388**	0.197	P63258, P11017, P62871	GNB2, GNB1, ACTG1
Thyrotropin-releasing hormone receptor signaling pathway (P04394)	2	0.082	0.759	P11017, P62871	GNB2, GNB1
FGF signaling pathway (P00021)	2	**0.0417**	0.2	P63103, P68250	YWHAZ,YWHAB
EGF receptor signaling pathway (P00018)	4	**0**	**0.041**	P63103, Q95115, P68250, Q9TTC5	YWHAZ, STAT5A, YWHAB, BTC
PI3 kinase pathway (P00048)	3	**0.002**	**0.0685**	P63103, P11017, P62871	GNB2, GNB1, YWHAZ
Opioid prodynorphin pathway (P05916)	2	**0.00406**	**0.0602**	P11017, P62871	GNB2, GNB1
Histamine H1 receptor mediated signaling pathway (P04385)	2	**0.00647**	**0.0502**	P11017, P62871	GNB2, GNB1
Enkephalin release (P05913)	2	**0.00387**	**0.0701**	P11017, P62871	GNB2, GNB1
Angiotensin II-stimulated signaling through G proteins and beta-arrestin (P05911)	2	**0.00488**	**0.0497**	P11017, P62871	GNB2, GNB1
CCKR signaling map (P06959)	2	0.0816	0.36	P62871, P68250	GNB1,YWHAB
Metabotropic glutamate receptor group II pathway (P00040)	2	**0.00647**	**0.0527**	P11017, P62871	GNB2, GNB1
Jersey Cattle					
Integrin signalling pathway (P00034)	2	0.0879	0.796	P62998, Q2TBH2	RAC1, RRAS
EGF receptor signaling pathway (P00018)	3	**0.00663**	0.36	P62998, Q2TBH2, Q95115	RAC1, RRAS, STAT5A
PDGF signaling pathway (P00047)	2	0.0567	0.66	Q148J4, Q95115	RAB2A, STAT5A
Blood coagulation (P00011)	3	**0.000428**	**0.0698**	P34955, P41361, P28800	SERPINA1, SERPINC1, SERPINF2
CCKR signaling map (P06959)	2	0.0872	0.836	P17697, P62998	CLU, RAC1
T cell activation (P00053)	2	**0.0318**	0.648	P01888, P62998	B2M, RAC1
TGF-beta signaling pathway (P00052)	2	**0.0298**	0.694	Q2TBH2, P21214	RRAS, TGFB2

## Discussion

The present study was designed to characterize and compare the milk proteome of Kashmiri and Jersey cattle. Over the past few decades, interest to reveal the dynamics of milk proteome has grown and there have been remarkable developments in the techniques used for fractionation and identification of proteins [25–27]. In the present study, a combination of fractionation and mass spectrometry techniques were used to comprehensively characterize the milk proteome profiles of Kashmiri and Jersey cattle breeds.

A total of 180 proteins were found to be differentially expressed between Kashmiri and Jersey cattle. Interestingly, 90 and 89 of the differentially expressed proteins were significantly upregulated in Kashmiri and Jersey cattle, respectively. Enzyme modulators were the major class of up-regulated proteins in both Kashmiri (20.51%) and Jersey cattle (14.28%). Hydrolases represented 12.82 and 14.28% of upregulated proteins in Kashmiri and Jersey cattle, respectively. Interestingly, chaperone class of proteins was only observed in milk of Kashmiri cattle. Chaperones help in the folding of newly synthesized proteins and prevent their premature (mis) folding at least until a domain capable of forming a stable structure is synthesized. As expected and in agreement with earlier studies ([26, 27]), the casein and whey fraction proteins were highly expressed in both breeds. However, a different set of high abundant milk proteins were significantly upregulated in each of the breeds. For example, the abundantly expressed proteins beta-casein, lactoferrin and CD4 were significantly upregulated in Kashmiri while beta-lacto globulin, kappa-casein and alpha-lactalbumin were significantly upregulated in Jersey (Table 1). Interestingly, the low abundant proteins FMO3, GLYCAM1, CD9, APLN, BTC, enterotoxin-binding glycoprotein PP16K, ORM1, serin peptidase inhibitor clade A, adipocyte differentiation-related protein and uterine milk protein were significantly upregulated in Kashmiri while ATP synthase subunit A, RAC1, B2M, SAR1B, TCN2 and MFGE8 were upregulated in Jersey. These results indicate a clear distinction as well as wide differences in the proteome profiles between the breeds which could be explained by high selection pressure for milk production traits in Jersey.

The differences in the expression of high abundant proteins between the breeds might confer differential benefits to their milks. For example, different levels of phosphorylation of beta-casein has been reported to affect the availability of calcium and protein micelle stability of milk [28], which might have important consequences on the nutrition and technological properties of milk and milk products. Additionally, other key bioactive proteins identified in this study that are well known to exert beneficial effects on human nutrition and health include lactoferrin, GLYCAM1, betacellulin, apelin, LALBA and serine peptidase inhibitor, etc. Iron sequestering properties of lactoferrin (LF), along with blockade of microbial carbohydrate metabolism and destabilisation of the bacterial cell wall [29, 30], has been shown to produce bactericidal and bacteriostatic effects in a wide range of microorganisms, including gram positive and gram negative bacteria, aerobes, anaerobes, yeasts and parasites [31–33]. Similarly, GLYCAM1 with a 7.93-fold expression in Kashmiri cattle is known to act as an antimicrobial peptide with ability to protect the intestinal mucosal tract of neonates largely due to its lubricating properties [34, 35]. In addition to these, apelin peptides might be involved in maturation of the gastrointestinal tract [36, 37]. Betacellulin (BTC), a key epidermal growth factor (EGF) [38] might regulate the development and maturation of the neonatal gut and immune system [39]. EGFs are major growth promoting factors in human milk [40] but the biological significance of BTC in bovine milk is currently unclear and needs further investigation. However, one plausible explanation for the presence of BTC in bovine milk might be to stimulate the proliferation of the gastrointestinal epithelia in new-borns, as has been proposed for milk-borne EGF and TGF-α (Transforming growth factor alpha) in other species [41]. With respect to Jersey breed, peptides resulting from partial digestion of high abundant proteins such as LALBA, CSN2 and CSN3 in the small intestine may influence gut functions including immune stimulation, mineral and trace element absorption and host defence against infection [42]. Alpha-lactalbumin enhances infant gastrointestinal function [43], motility and antimicrobial activity [44]. CSN3 is readily hydrolysed in calf’s stomach, allowing the formation of a coagulum that can be readily digested [45] and also provides heat stability to milk by stabilising the casein micelle [45]. Moreover, CSN3 prevents infection by disrupting the attachment of pathogens to mucosal cells [46]. CSN3 digestion results in the formation of a glycomacropeptide which in turn enhances mineral absorption [47]. Bovine beta 2-microglobulin (B2M) is an antibacterial protein present in milk fat globules. B2M possesses potent antibacterial activities against Gram positive pathogenic bacteria [48]. Bovine milk is an abundant source of bioavailable B12 vitamin wherein when complexed with transcobalamin, a major vitamin B12 binding protein in cows’ milk [49], stimulates vitamin B12 absorption through intestinal epithelial cells [50]. Lactadherin is secreted by mammary epithelial cells and stored in milk fat globules [51]. Lactadherin, as one of the immune components in bovine milk has been found to prevent rota viral infection in infants by removing the sialic acid from the viral coat [52, 53].

It is worthwhile to note that the low abundant protein, flavin-containing monooxygenase 3 (FMO3) had 16.6 fold expression rate in Kashmiri as compared to Jersey. This is the first report wherein FMO3 has been found to be highly expressed in Kashmiri cattle. Increased presence of FMO3 might be important due to its ability to oxidise trimethylamine (TMA), a compound with fishy odour, to TMAO (Trimethylamine N-oxide), an odourless oxide. Absence of FMO3 leads to fishy flavour in milk due to increased build-up of TMA, and thus might play an important role in maintaining the quality of milk [54–56]. Moreover, FMO3 belongs to a drug metabolising enzyme class with ability to oxidize xenobiotics, pesticides and other foreign inhabitants in body fluids including milk and serum [57–60] and hence presents an efficient defence mechanism in new-borns. The presence of FMO3 at high concentrations in Kashmiri cattle milk can favour utilization of Kashmiri cattle milk in commercial preparations for the promotion of human health and nutritional status. In fact, bio-mining of such bioactive milk protein constituent and marketing it as ingredients may not only serve as a lucrative business for the Indian dairy industry but also in the development of products for consumers with special needs like allergy and milk tolerance.

The GO analysis of significantly up-regulated proteins revealed only one significantly enriched GO term (extracellular region) after FDR correction in both breeds and limited functional overlap was found between the present proteomic data and our earlier transcriptome data [61] indicating the failure of RNA-based analyses to represent completely protein dynamics [62].

Pathway analysis helps in biological interpretation of proteomic and other high-throughput data in cells or organisms [63]. Most of the pathways (Wnt signaling pathway, EGF receptor signaling pathway, FGF signaling pathway, PI3 kinase pathway) significantly enriched by the significantly upregulated proteins in Kashmiri cattle are involved in mammary gland development. Wnt signaling pathway regulates mammary development [64] during various stages of mammary morphogenesis [65]. The proteins enriched in the Wnt signalling pathway were GNB1(G protein subunit beta 1), GNB2 (G protein subunit bBeta 2) and ACTG1(actin gamma 1). ACTG1 plays a critical role in branching and alveolar development of the mammary gland through cytoskeletal remodelling [66]. FGF signalling pathway controls mammary epithelial cell branching and morphogenesis [67] and activates PI3 kinase pathway through phosphorylation [68]. Epidermal growth factor family plays essential roles in regulating cell proliferation, survival and differentiation of mammary epithelial cells through STAT5A, a key non-tyrosine kinase protein indirectly regulated by JAK2/ELF5, insulin growth factor, estrogen, and progesterone signalling pathways [69]. In Jersey cattle, two significantly (*p* < 0.05) enriched pathways, blood coagulation/coagulation cascades and T cell activation pathways are associated with immune system regulation [70]. *SERPINA1*, *SERPINC1*, *SERPINF2* are important proteins in blood coagulation pathway whereas, *B2M* and *RAC1* play critical roles in T cell activation pathway. These proteins play fundamental roles in innate immunity in addition to enhancing adaptive immune responses [71]. Altogether, a wide range of proteins were detected in this study including proteins involved in immune response, host defense and milk quality as well as qualitative and quantitative differences in their milk proteome.

## Conclusion

A total of 91 and 89 proteins were significantly upregulated in Kashmiri and Jersey cattle, respectively. A different set of high- abundant and low-abundant proteins were significantly upregulated in Kashmiri and Jersey cattle, clearly differentiating the two breeds at the proteome level. Immune-related proteins (CD4, LF and GLYCAM 1) and drug metabolising enzyme (FMO3) were abundantly expressed in Kashmiri cattle milk. The presence of FMO3 at high concentrations in Kashmiri cattle milk could favour its utilization in commercial preparations for human health promotion and consequently serve as a boost for increased business opportunities for the Indian dairy industry.

## Methods

### Experimental animals and sampling

The ethical clearance was approved by the Institutional Animal Ethics Committee (IAEC) of Sher-e-Kashmir University of Agricultural Sciences and Technology of Kashmir. A total of three healthy Kashmiri and three Jersey cows in their 3rd lactation from the university dairy farm (Mountain Livestock Research Institute, Share-Kashmir University of Agricultural Sciences and Technology of Kashmir, India) were selected for the study. The animals were kept under similar feeding and management conditions to minimise environmental variation. Fresh milk samples (200 mL) were aseptically collected from all the four quarters (50 mL per quarter) at day 90 in milk (D90), mixed thoroughly, placed on ice and immediately transported to the laboratory for further analysis.

### Protein preparation

Milk samples were processed differently for high and low abundance protein analysis. For high-abundance protein analysis, 50 mL of milk was immediately placed on ice after collection followed by centrifugation at 4000×g for 10 min at 4 °C within 2 h of collection. The fat layer was removed and skimmed fraction was stored at − 20 °C. Whereas, for low abundance protein analysis, 0.24 mL (100X) mammalian protease inhibitor cocktail (Sigma, Milwaukee, WI, USA) was added to 50 mL of milk followed by centrifugation at 4000×g for 15 min at 4 °C. The cream layer was removed and the skimmed or whey portion was depleted of casein using a previously described method [72]. Briefly, 60 mM CaCl2 was added to skimmed sample and the pH was adjusted to 4.3 using 30% acetic acid (Fisher Scientific, Fair Lawn, NJ, USA). Samples were then centrifuged at 189,000×g at 4 °C for 70 min and the supernatant was collected and stored at − 80 °C.

### Enrichment of low abundance proteins

Low abundance minor proteins were enriched using the ProteoMiner Kit (BioRad Laboratories, Hercules, CA, USA) as per manufacturer’s protocol. Whey samples were placed in individual ProteoMiner columns, mixed thoroughly by shaking (gently) followed by incubation at room temperature for 2 h. Subsequently, samples were washed thoroughly using HPLC grade water to remove excess proteins by centrifugation at 7000 g for 5 min. Low abundance proteins were eluted off the beads by addition of 20 μl 4 x Laemmli sample buffer (8% SDS, 40% glycerol, 250 mM Tris, pH 6.8, 400 mM DTT with trace amount of bromophenol blue).

### In-solution digestion of proteins and nano-scale LC/MS analysis on QTOF

The pellets after acetone precipitation (high abundant proteins) or TCA (Trichloroacetic acid)-acetone precipitation (low abundant proteins) were dissolved in 50 mM ammonium bicarbonate (dilution 1:3) and 0.1% SDS. 100 μg of the extracted protein was subjected to in solution trypsin digestion with carbamidomethylation at cysteine (fixed) and oxidation at methionine (variable). The dissolved pellet was treated with 10 μl of 100 mM DTT (Dithiothreitol) followed by incubation on a thermo mixer (Eppendorf ThermoMixer® C,) at 95 °C for 1 h. The sample was treated with 18 μl of 250 mM IDA (Iodoacetamide) and then incubated in the dark for 45 min at room temperature. To stop the IDA reaction, 40 μl DTT was added at room temperature and incubated for 10 min. To this solution, 50 mM ammonium bicarbonate and 0.1% SDS was added to make up the volume to 300 μl. For Enzymatic cleavage of the protein, trypsin in the ratio 50:1 (w/v) was added to sample and incubated on the thermo mixer at 37 °C overnight. To stop the trypsin activity, the peptides were then extracted in 0.1% formic acid followed by incubation at 37 °C for 45 min. The extracted mixture was then centrifuged at 13000 g for 10 min and the supernatant was placed in a separate Eppendorf tube. This supernatant was subjected to speed vac at 45 °C. The resulting peptides were then dissolved in 20 μl of 0.1% formic acid and 10 μL of this solution was used on C18 UPLC column for separation of peptides. The mass spectrometer was operated in positive ion mode, and MS spectra were acquired over a range of 375–1500 m/z. For MS and MS/MS scans, the resolution of the orbitrap fusion was set at 120,000 and 50,000 at 200 m/z, respectively. Data-dependent acquisition mode was set as top speed, and ions were fragmented (10 fragment files collected after every full scan) through higher energy collisional dissociation, and cycle time was 3 s with peptide mass tolerance and fragment mass tolerance of 50 ppm and 100 ppm, respectively. The automatic gain control target values for master scan modes and MS/MS were set to 4e^5^ and 1e^5^, respectively. Dynamic exclusion duration was 40 s.

### Protein identification and differential expression analysis

The individual peptides MSMS spectra were searched against the Swiss-Prot databases using the Mascot Distiller Search engine (v. 2.6.0) for protein identification and expression analysis was performed with PLGS software (Protein Lynx Global Server, Waters, India) by Sandor’s Lifesciences, Hyderabad, India. The results were filtered based on the peptide Benjaminin and Hochberg corrected *p*-value < 0.1 (FDR < 0.1) or uncorrected p-value < 0.05. Both unique and razor peptides were selected for protein quantification, protein ratios were calculated as the median of only unique or razor peptides of the protein. All peptide ratios were normalized based on the median ratio. The protein species quantification results were statistically analysed by student’s t-test, and the p-value was corrected by the method of Benjamin and Hochberg FDR analysis. An FDR < 0.1 was considered significant due to the low number of samples analysed.

### Gene ontology and pathway analysis

Gene ontology (GO) and pathway enrichment analysis of differentially expressed proteins was accomplished with Gene Ontology Consortium data base (http://www.geneontology.org) (Falcon and Gentleman, 2007). GO terms and KEGG pathways (http://www.genome.jp/kegg/) with FDR < 0.1were considered significantly enriched.

## Supplementary information

**Additional file 1 : Table S1.** Significantly upregulated proteins in Kashmiri cattle (high and low abundant proteins) **Table S2.** Significantly upregulated proteins in Jersey cattle (high and low abundant proteins). **Table S3.** Sample chromatograms of individual samples. **Table S4-S6.** Peptide information of significantly upregulated proteins in Jersey cattle. **Table S7-S9.** Peptide information of significantly upregulated proteins in Kashmiri cattle.

**Additional file 2 : Table S10.** Classification of significantly upregulated proteins in Kashmiri and Jersey cattle.

**Additional file 3.** Total proteome obtained from different samples.

## Data Availability

The datasets generated and analysed during the current study are available as Additional files.

## Abbreviations

AGP: α-1-acid glycoprotein
APLN: Apelin
B2M: Beta 2-microglobulin
BTC: Betacellulin
CSN2: Beta-casein
CSN3: Kappa-casein
CYM: Chymosin
EGF: Epidermal growth factor
EGR1: Early growth response protein 1
EHD: EH domain-containing protein 1
FDR: False discovery rate
FGF: Fibroblast growth factor
FMO3: Flavin mono-oxygenase3
GALNT1: Polypeptide N-Acetylgalactosaminyltransferase
GLYCAM1: Glycosylation-dependent cell adhesion molecule 1
GO: Gene ontology
HSP90AA1: Heat shock protein90AA1
LALBA: Alpha-lactalbumin
LC-MS/Q-TOF: Liquid chromatography-mass spectrometry/quantitative time of flight
LF: Lactoferrin
LGB: Beta-lactoglobulin
LPL: Lipoprotein lipase
LTF: Lactotransferrin
MEC: Mammary epithelial cell
PNLIPRP2: Pancreatic lipase related protein 2
RAC1: Ras-related C3 botulinum toxin substrate 1
SERPINA1: Serine protease inhibitor1
TGF-α: Transforming growth factor
TLR2: Toll like receptor 2
TMAO: Trimethylamine N-oxide
ZNF496: Zinc finger protein 496
